# Balancing Bleeding and Ischemic Risks: A Systematic Review of Dual Versus Triple Therapy After Percutaneous Coronary Intervention in Patients With Atrial Fibrillation

**DOI:** 10.7759/cureus.99187

**Published:** 2025-12-14

**Authors:** Abubakar Gapizov, Ahmad Mohammad, Shivam Singla, Bhavna Singla, Saifullah Syed, Sunita Kumawat, Zulqurnain Ali

**Affiliations:** 1 Internal Medicine, NewYork-Presbyterian Brooklyn Methodist Hospital, New York, USA; 2 Internal Medicine, Hurley Medical Center, Flint, USA; 3 Internal Medicine, TidalHealth Penninsula Regional, Salisbury, USA; 4 Internal Medicine, Erie County Medical Center Health Campus, Buffalo, USA; 5 Internal Medicine, Royal College of Surgeons in Ireland, Dublin, IRL; 6 Internal Medicine, St. Francis Medical Center, Lynwood, USA; 7 Internal Medicine, Rawalpindi Medical University, Rawalpindi, PAK

**Keywords:** atrial fibrillation, bleeding risk, dual antithrombotic therapy, ischemic outcomes, non-vitamin k oral anticoagulants, percutaneous coronary intervention, triple therapy

## Abstract

This systematic review evaluates the comparative efficacy and safety of dual versus triple antithrombotic therapy in patients with atrial fibrillation undergoing percutaneous coronary intervention. A comprehensive literature search identified four randomized controlled trials encompassing diverse populations and treatment strategies. Dual therapy, consisting of a non-vitamin K oral anticoagulant combined with a single P2Y12 inhibitor, consistently demonstrated a reduction in bleeding complications compared to conventional triple therapy without compromising protection against ischemic events such as myocardial infarction, stroke, or stent thrombosis. While the larger multicenter trials provided robust evidence supporting the safety of early aspirin withdrawal, smaller trials were limited by sample size and early termination, restricting the generalizability of their findings. Nevertheless, the overall evidence suggests that dual therapy should be considered the preferred approach for most patients, with triple therapy reserved for carefully selected individuals at high ischemic risk where a short course may provide incremental benefit. These findings support the growing shift in clinical practice toward individualized antithrombotic strategies that optimize both safety and efficacy in this complex patient population.

## Introduction and background

Atrial fibrillation (AF) is the most common sustained cardiac arrhythmia, affecting millions worldwide and conferring a substantially increased risk of stroke and systemic embolism. Systemic embolism refers to the obstruction of blood flow in arteries outside the heart and brain by a clot originating from the heart. Oral anticoagulation (OAC) is the cornerstone of therapy for stroke prevention in patients with AF [1]. However, a significant proportion of patients with AF also have concomitant coronary artery disease (CAD) requiring percutaneous coronary intervention (PCI) and stent placement [2]. Stent placement involves the insertion of a small metal scaffold to keep a narrowed coronary artery open. In this setting, patients not only require anticoagulation for prevention of cardioembolic events but also antiplatelet therapy to reduce the risk of stent thrombosis and recurrent ischemic events [3]. Stent thrombosis is the acute formation of a blood clot within the stent, which can lead to myocardial infarction or sudden cardiac death.

Traditionally, the combination of OAC with dual antiplatelet therapy (aspirin plus a P2Y12 inhibitor) - commonly referred to as triple antithrombotic therapy (TTT) - was considered the standard of care for patients with AF undergoing PCI [4]. P2Y12 inhibitors are a class of antiplatelet drugs (such as clopidogrel) that prevent platelet activation and aggregation. While effective in reducing ischemic complications, TTT has consistently been associated with a markedly elevated risk of major and clinically significant bleeding. This increased bleeding risk arises from the simultaneous inhibition of multiple pathways of hemostasis, as anticoagulants suppress the coagulation cascade while dual antiplatelet agents inhibit platelet function, resulting in an additive or synergistic hemorrhagic effect. This risk is particularly concerning in elderly patients and those with comorbidities such as renal dysfunction or diabetes, who represent a substantial subset of the AF-PCI population [5].

To address the bleeding hazard associated with TTT, the concept of dual antithrombotic therapy (DAT) emerged. DAT refers to the use of one oral anticoagulant combined with only a single antiplatelet agent instead of two. DAT typically involves an oral anticoagulant (either a vitamin K antagonist or, more recently, a direct oral anticoagulant (DOAC)) combined with a single antiplatelet agent, most often clopidogrel [6]. Several pivotal randomized controlled trials (RCTs) over the last decade have tested DAT against TTT, evaluating both safety and efficacy in reducing bleeding and thromboembolic outcomes [7].

The objective of this systematic review is to compare the efficacy and safety of DAT versus TTT in patients with AF undergoing PCI, focusing on evidence from pivotal RCTs. By synthesizing data from these key studies, this review aims to clarify the optimal strategy that balances protection against thromboembolic and ischemic events while minimizing bleeding risk.

## Review

Materials and methods

Search Strategy

This systematic review was conducted in accordance with the Preferred Reporting Items for Systematic Reviews and Meta-Analyses (PRISMA) guidelines [8]. A comprehensive literature search was performed across PubMed, Embase, Scopus, and the Cochrane Central Register of Controlled Trials from database inception until September 2025. The search strategy combined Medical Subject Headings (MeSH) and free-text terms related to “atrial fibrillation,” “percutaneous coronary intervention,” “dual therapy,” “triple therapy,” “oral anticoagulants,” and “randomized controlled trial.” Reference lists of relevant reviews and included articles were hand-searched to identify additional eligible studies. Only peer-reviewed studies published in English were considered.

Eligibility Criteria

The inclusion and exclusion process was guided by a PICO framework [9]. The population of interest included adult patients with atrial fibrillation (AF) undergoing percutaneous coronary intervention (PCI) with stent placement. The intervention was defined as DAT consisting of a non-vitamin K oral anticoagulant (NOAC) or vitamin K antagonist (VKA) combined with a single P2Y12 inhibitor, without routine aspirin use. The comparator was conventional triple therapy, defined as an oral anticoagulant combined with dual antiplatelet therapy, typically aspirin plus a P2Y12 inhibitor. The primary outcomes of interest were bleeding events, including major and clinically relevant nonmajor bleeding, while secondary outcomes included ischemic events such as myocardial infarction, stroke, systemic embolism, cardiovascular death, stent thrombosis, and unplanned revascularization. Eligible studies were RCTs that directly compared dual and triple therapy. Observational studies, registry analyses, case reports, and non-comparative trials were excluded.

Study Selection and Data Extraction

Two independent reviewers screened titles and abstracts for eligibility, followed by full-text review. Discrepancies were resolved through consensus with a third reviewer. Data were extracted using a predesigned template that captured study characteristics, sample size, inclusion criteria, intervention and comparator details, follow-up duration, and outcome measures. Extracted outcomes included both efficacy endpoints and bleeding events, consistent with standardized definitions such as those from the Bleeding Academic Research Consortium (BARC) and Thrombolysis in Myocardial Infarction (TIMI) classifications when available.

Risk of Bias Assessment

The methodological quality of included trials was evaluated using the Cochrane Collaboration’s Risk of Bias 2 (RoB 2) tool [10]. Domains assessed included randomization process, allocation concealment, blinding of participants and outcome assessors, completeness of outcome data, selective reporting, and other potential sources of bias. Each domain was graded as “low risk,” “some concerns,” or “high risk.” Risk of bias judgments were performed independently by two reviewers, with consensus reached through discussion.

Data Synthesis

Given the heterogeneity in interventions, comparators, and outcome reporting across the included trials, a narrative synthesis was performed. Results were structured to provide a comparative analysis of dual versus triple therapy across pivotal RCTs. Where possible, hazard ratios and 95% confidence intervals reported by the original studies were summarized to facilitate comparison of treatment effects. The overall quality of evidence was considered in light of trial design, sample size, and consistency of findings.

Results

Study Selection Process

**Figure 1:**
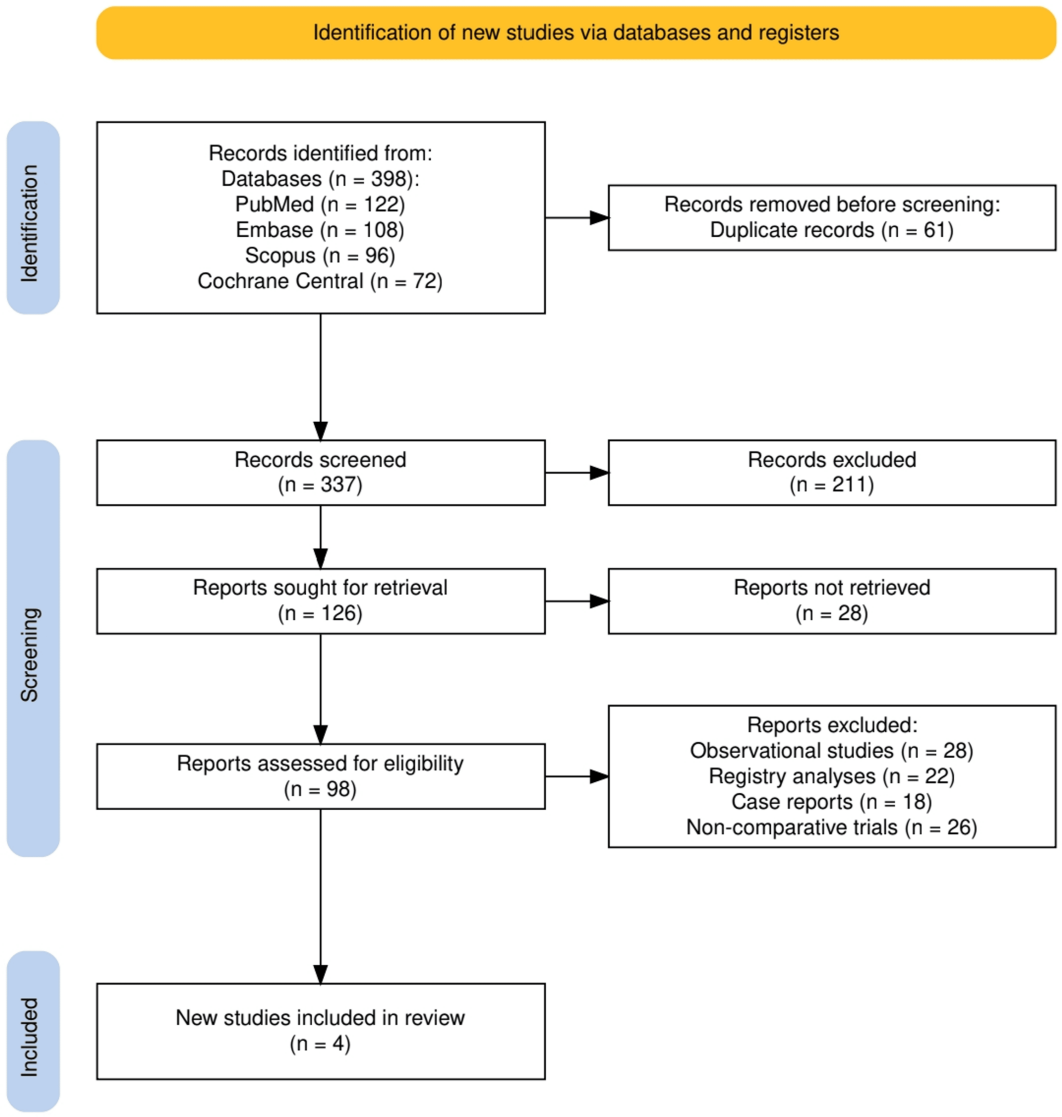
The PRISMA flow diagram represents the study selection process. PRISMA, Preferred Reporting Items for Systematic Reviews and Meta-Analyses

The study selection process followed PRISMA guidelines and is outlined in Figure 1. A total of 398 records were identified through database searching, including PubMed (*n* = 122), Embase (*n* = 108), Scopus (*n* = 96), and Cochrane Central (*n* = 72). After removal of 61 duplicates, 337 records were screened, of which 211 were excluded based on title and abstract review. Of the 126 full-text articles assessed for eligibility, 28 were not retrievable and 98 were reviewed in detail. A further 94 articles were excluded, primarily due to being observational studies (*n* = 28), registry analyses (*n* = 22), case reports (*n* = 18), or non-comparative trials (*n* = 26). Ultimately, four RCTs met all inclusion criteria and were incorporated into the final analysis.

Characteristics of the Selected Studies

The key characteristics of the RCTs included in this review are summarized in Table 1. Collectively, these studies enrolled patients with AF undergoing PCI and compared various DAT regimens with TTT. Sample sizes ranged from over 2,700 participants in the largest trial to just above 100 in smaller, more population-specific studies, highlighting differences in statistical power and generalizability. Interventions primarily included non-vitamin K oral anticoagulants in combination with a P2Y12 inhibitor, while comparators used vitamin K antagonists plus dual antiplatelet therapy. Follow-up durations were consistently one year, and primary outcomes focused on bleeding events, with secondary measures capturing ischemic outcomes such as myocardial infarction, stroke, or stent thrombosis. Despite variations in trial design and patient populations, the overarching findings revealed that dual therapy substantially reduced bleeding risks without compromising efficacy in preventing thromboembolic complications, though smaller trials were underpowered to draw definitive conclusions.

**Table 1:** Characteristics of randomized controlled trials comparing dual and triple antithrombotic therapy after PCI in atrial fibrillation. AF, atrial fibrillation; PCI, percutaneous coronary intervention; RCT, randomized controlled trial; NOAC, non–vitamin K antagonist oral anticoagulant; VKA, vitamin K antagonist; DAPT, dual antiplatelet therapy; DAT, dual antithrombotic therapy; TTT, triple antithrombotic therapy; MI, myocardial infarction; HR, hazard ratio; CI, confidence interval; BARC, Bleeding Academic Research Consortium; TIMI, Thrombolysis in Myocardial Infarction

Study (Author, Year)	Design	Population (N, key inclusion criteria)	Intervention (dual therapy)	Comparator (triple therapy)	Follow-up duration	Primary outcomes	Key findings
Kerneis et al. (2019) (PIONEER AF-PCI subanalysis) [11]	RCT	2,124 patients with AF undergoing PCI; stratified by INR stability in the VKA group	Rivaroxaban 15 mg OD + P2Y12 inhibitor (*n *= 709) OR rivaroxaban 2.5 mg BID + DAPT (*n *= 709)	Warfarin + DAPT (*n *= 706)	12 months	Clinically significant bleeding	Rivaroxaban-based regimens significantly reduced bleeding compared with VKA + DAPT, regardless of INR control (HR range 0.42-0.76, *P *< 0.05)
Cannon et al. (2017) (RE-DUAL PCI) [12]	RCT	2,725 patients with AF post-PCI; randomized to dabigatran 110 mg or 150 mg BID + P2Y12 inhibitor (clopidogrel/ticagrelor) vs warfarin-based TTT	Dabigatran (110 mg or 150 mg BID) + P2Y12 inhibitor (no aspirin)	Warfarin + P2Y12 inhibitor + aspirin (1-3 months)	Mean 14 months	Major or clinically relevant nonmajor bleeding; composite efficacy endpoint (MI, stroke, systemic embolism, death, or revascularization)	DAT significantly reduced bleeding vs. TTT (HR 0.52 for 110 mg, HR 0.72 for 150 mg); DAT was noninferior to TTT for ischemic outcomes (HR 1.04; 95% CI 0.84-1.29)
Hoshi et al. (2020) (SAFE-A) [13]	RCT	210 patients with AF requiring PCI with stenting; mean age 72.7 ± 8.2 years, 81% male	Apixaban + aspirin (after 1 month P2Y12 discontinuation)	Apixaban + aspirin + P2Y12 inhibitor for 6 months	12 months	Any bleeding event (TIMI, BARC, or requiring transfusion)	No significant difference in bleeding between groups (11.8% vs. 16.0%; HR 0.70, 95% CI 0.33-1.47, *P *= 0.35). The trial was underpowered due to early termination of enrollment.
Liu et al. (2021) (Contemp Clin Trials) [14]	RCT	106 elderly Chinese patients with NVAF post-PCI	Rivaroxaban 15 mg OD + Ticagrelor 90 mg BID	Warfarin (dose-adjusted) + Aspirin 100 mg OD + Clopidogrel 75 mg OD	12 months	Composite of CV death, MI, stroke, or stent thrombosis; Safety: clinically significant bleeding	No significant difference in efficacy (16.7% vs. 15.2%, HR 1.02, 95% CI 0.82-1.24, *P *= 0.86). Dual therapy had significantly lower bleeding risk (7.4% vs. 26.9%, HR 0.71, 95% CI: 0.62-0.83, *P *= 0.01).

Quality Assessment

The quality assessment of the included RCTs is presented in Table 2. Overall, the larger multicenter trials demonstrated low risk of bias across all domains, with appropriate randomization procedures, well-balanced groups, and rigorous use of standardized outcome definitions for both bleeding and ischemic events. Their open-label designs were mitigated by the use of objective and adjudicated endpoints, reducing the likelihood of systematic bias. In contrast, smaller studies presented some methodological concerns, particularly due to limited sample sizes, incomplete information regarding allocation concealment, and premature termination of recruitment, which increased the risk of imbalance and restricted the ability to fully validate prespecified outcomes. Despite these limitations, the consistency of objective outcome measurement across all trials strengthens confidence in the overall findings, although the evidence from underpowered studies should be interpreted cautiously within the broader context of the more robust trials.

**Table 2:** Risk-of-bias assessment of included randomized controlled trials. RoB 2, Cochrane Risk of Bias 2 tool; RCT, randomized controlled trial; AF, atrial fibrillation; PCI, percutaneous coronary intervention

Study (author, year)	Randomization process	Deviations from intended interventions	Missing outcome data	Measurement of outcomes	Selection of reported results	Overall risk of bias
Kerneis et al. (2019) (PIONEER AF-PCI subanalysis) [11]	Low risk - randomization appropriate, balance maintained across groups	Low risk - open-label design, but objective bleeding endpoints reduce concern	Low risk - follow-up was adequate, minimal missing data	Low risk - standardized definitions (TIMI, ISTH bleeding)	Low risk - outcomes reported as per protocol	Low risk
Cannon et al. (2017) (RE-DUAL PCI) [12]	Low risk - multicenter randomization with stratification	Low risk - open-label, but the primary outcome (bleeding) is objective	Low risk - dropout rate balanced and handled appropriately	Low risk - adjudicated events, standardized outcome definitions	Low risk - outcomes consistent with published protocol (NCT registry)	Low risk
Hoshi et al. (2020) (SAFE-A) [13]	Some concerns - early termination of trial → possible imbalance in allocation	Some concerns - underpowered, small sample size increases bias risk	Low risk - follow-up adequate for enrolled patients	Low risk - objective bleeding measures used	Some concerns - early termination limited the ability to confirm prespecified outcomes	Some concerns
Liu et al. (2021) (Contemp Clin Trials) [14]	Some concerns - relatively small sample size, unclear details on randomization concealment	Low risk - interventions applied as planned	Low risk - complete follow-up achieved (1 year)	Low risk - objective, clinically adjudicated endpoints	Some concerns - limited protocol information, selective reporting possible	Some concerns

Discussion

Efficacy and Safety of Dual Versus Triple Therapy

Our review of pivotal RCTs evaluating antithrombotic strategies in patients with AF undergoing PCI highlights a consistent and clinically meaningful finding: DAT, consisting of a non-vitamin K antagonist oral anticoagulant (NOAC) plus a single antiplatelet agent, significantly reduces bleeding complications without sacrificing protection against ischemic events when compared to traditional triple therapy (TTT) regimens. Across large-scale trials such as RE-DUAL PCI [12] and PIONEER AF-PCI [11], DAT was associated with a 40-50% relative reduction in major or clinically relevant non-major bleeding, with hazard ratios consistently ranging between 0.52 and 0.72, depending on drug regimen and dosing strategies. Importantly, these reductions in bleeding did not come at the cost of increased rates of thromboembolic complications, myocardial infarction, or stent thrombosis, with dual therapy demonstrating noninferior efficacy to triple therapy across diverse patient populations and follow-up periods. Even smaller studies, such as SAFE-A [13] and Liu et al.’s Chinese RCT [14], confirmed the same direction of effect, showing a trend toward lower bleeding and comparable ischemic outcomes despite limited statistical power. Taken together, these findings consolidate the emerging paradigm that the inclusion of aspirin in prolonged regimens offers little incremental ischemic benefit but substantially magnifies bleeding risk, thereby tilting the balance of risk and benefit decisively in favor of DAT in the majority of AF patients requiring PCI.

Alignment With Contemporary Guidelines and Real-World Data

These results are highly consistent with contemporary international guidelines and extend the growing body of evidence that has redefined antithrombotic strategies in AF patients undergoing PCI over the past decade. The 2020 European Society of Cardiology (ESC) guidelines explicitly recommend minimizing the duration of triple therapy to the shortest feasible period, often limited to one week or up to one month in select high-ischemic-risk individuals, before transitioning to DAT [15]. Similarly, the 2019 American Heart Association/American College of Cardiology/Heart Rhythm Society (AHA/ACC/HRS) update emphasizes NOAC-based dual therapy as the default strategy, reserving triple therapy for exceptional cases [16]. Our findings reinforce these recommendations, providing trial-level evidence that aligns with the risk-benefit calculus outlined in guideline statements. Moreover, large meta-analyses pooling over 10,000 patients across multiple RCTs have demonstrated that DAT reduces major bleeding by nearly 50% (risk ratio ~0.52) while maintaining comparable rates of death, myocardial infarction, and stroke relative to TTT [17]. Real-world registry data, including analyses from the Danish nationwide cohort and the U.S. National Cardiovascular Data Registry, echo these findings, underscoring their external validity in routine practice [18]. However, registry studies have also revealed that clinicians continue to prescribe triple therapy more liberally than guidelines suggest, often driven by concern over stent thrombosis in complex PCI cases. This disconnect between evidence, guidelines, and practice highlights the critical role of systematic reviews like ours in reinforcing the evidence base, bridging knowledge gaps, and encouraging a more nuanced, patient-centered approach to balancing ischemic protection against bleeding risk in this high-stakes population.

Patient Subgroups, Drug-Specific Considerations, and Trial Nuances

A closer examination of these pivotal trials reveals nuances that extend beyond the binary comparison of dual versus triple therapy and underscore the importance of patient and drug-specific considerations. Subgroup analyses suggest that elderly patients, those with impaired renal function, and individuals with diabetes may particularly benefit from NOAC-based dual therapy due to their heightened susceptibility to bleeding complications [19,20]. Yet, the safety profile of DAT must be weighed against ischemic risks in subsets such as those presenting with ST-elevation myocardial infarction (STEMI), undergoing complex PCI, or requiring multiple stents, where even a short duration of aspirin may provide additive protection. Drug-specific insights are also essential: rivaroxaban, dabigatran, and apixaban were tested in different dosing strategies, with rivaroxaban used at reduced doses in PIONEER AF-PCI [11] and dabigatran studied at both 110 mg and 150 mg BID in RE-DUAL PCI [12]. While these regimens reduced bleeding consistently, the lack of uniformity in trial design complicates direct comparisons between NOACs. Methodological considerations further temper the interpretation of results: SAFE-A [13] was terminated prematurely and remains hypothesis-generating, while Liu et al.’s trial, though suggestive, was limited to a small elderly Chinese cohort, restricting external validity. Even the larger, robust trials such as RE-DUAL PCI [12] and PIONEER AF-PCI [11] were open-label, introducing the potential for bias in reporting secondary outcomes. These insights highlight the need for nuanced interpretation and careful extrapolation, particularly in tailoring therapy to patients at the extremes of bleeding and ischemic risk.

From a clinical perspective, the accumulated evidence strongly supports a shift toward NOAC-based dual therapy as the default approach for most AF patients undergoing PCI. Patients with moderate bleeding risk and no extraordinary ischemic burden are the clearest beneficiaries, as DAT provides substantial reductions in major bleeding without sacrificing efficacy in preventing stroke, myocardial infarction, or stent thrombosis [21]. However, a short course of triple therapy may remain justifiable in carefully selected high-risk subsets, particularly those with extensive multivessel stenting, complex bifurcation lesions, or recent STEMI, where ischemic risk transiently outweighs bleeding concerns [22]. Clinical decision-making must be individualized, incorporating established risk stratification tools such as CHA₂DS₂-VASc for thromboembolic risk and HAS-BLED for bleeding risk [23], while also considering procedural complexity. The optimal choice of P2Y12 inhibitor remains unresolved; while clopidogrel was used predominantly in the large RCTs, real-world practice often favors ticagrelor or prasugrel in ACS patients, raising uncertainty about generalizability. Regional differences further complicate translation, as Asian populations have demonstrated higher bleeding rates with comparable or lower ischemic risk, suggesting that shorter durations of triple therapy-or even immediate DAT-may be especially advantageous in these patients. Thus, the practical implication of these findings is a call for individualized therapy, balancing ischemic and bleeding risk in a dynamic, patient-centered fashion rather than adhering rigidly to a one-size-fits-all strategy.

Limitations of Current Evidence and Gaps in Generalizability

Despite their transformative influence on practice, the current body of evidence is not without limitations. Most of the pivotal trials were open-label, which introduces the potential for performance and detection bias, even though the primary endpoints such as bleeding are largely objective. Several trials were either underpowered or prematurely terminated, such as SAFE-A [13], which compromises the precision of their findings and renders their results exploratory rather than definitive. Heterogeneity in drug regimens further complicates interpretation: while rivaroxaban, dabigatran, and apixaban all demonstrated favorable outcomes in DAT, differences in dosing schedules, background P2Y12 inhibitor selection, and variable use of aspirin preclude direct head-to-head comparisons. Furthermore, the trials primarily enrolled patients at moderate risk and excluded many with extreme profiles, leaving limited evidence for particularly vulnerable groups such as frail elderly, cancer patients, and those with high bleeding risk scores. In addition, while major contemporary trials have substantially contributed to the evidence base supporting dual therapy, they were not included in this review due to predefined selection criteria and study design considerations. Our review, although comprehensive, is constrained by these same limitations of the underlying evidence, and the absence of large-scale real-world validation in diverse populations remains an important caveat when applying these findings broadly to clinical practice.

Future Directions for Optimizing Antithrombotic Strategies

Future research must focus on refining antithrombotic strategies through more inclusive, precise, and comparative studies that address the gaps left by existing trials. Larger RCTs are needed in understudied populations such as Asian cohorts, who face disproportionate bleeding risk, elderly patients with multimorbidity, and those presenting with STEMI or undergoing high-complexity PCI. Direct head-to-head comparisons of different NOAC-based dual therapies, with standardized dosing and consistent background antiplatelet regimens, would clarify whether one approach confers superior safety or efficacy. The optimal duration of aspirin in high-ischemic-risk patients remains unresolved, and trials specifically designed to test varying durations of triple therapy in these populations could provide critical guidance. Moreover, advances in pharmacogenomics and biomarker discovery open the possibility for genetic or biomarker-guided antithrombotic tailoring, enabling clinicians to more accurately balance ischemic and bleeding risks at the individual level. Integration of these strategies with clinical risk scores may herald a new era of precision antithrombotic therapy, bridging the gap between population-level trial findings and the heterogeneity of real-world practice.

## Conclusions

Cumulative evidence from pivotal randomized trials demonstrates that DAT with a NOAC plus a P2Y12 inhibitor provides a safer and equally effective alternative to conventional triple therapy in patients with AF undergoing PCI. The consistent reduction in bleeding across diverse trial designs, coupled with noninferiority in ischemic outcomes, underscores a paradigm shift toward minimizing aspirin exposure after the peri-PCI period. While nuances remain for specific subgroups - such as patients with complex PCI or very high ischemic risk - the balance of current evidence strongly favors dual therapy as the default strategy, with triple therapy reserved for carefully selected cases and of limited duration. The take-home message is clear: for most patients with AF undergoing PCI, less is more - dual therapy reduces harm without compromising efficacy, and treatment should be tailored using individualized assessments of ischemic and bleeding risk.
